# Effect of Creosote Bush-Derived NDGA on Expression of Genes Involved in Lipid Metabolism in Liver of High-Fructose Fed Rats: Relevance to NDGA Amelioration of Hypertriglyceridemia and Hepatic Steatosis

**DOI:** 10.1371/journal.pone.0138203

**Published:** 2015-09-22

**Authors:** Haiyan Zhang, Yihang Li, Jie Hu, Wen-Jun Shen, Madhurima Singh, Xiaoming Hou, Alex Bittner, Stefanie Bittner, Yuan Cortez, Juveria Tabassum, Fredric B. Kraemer, Salman Azhar

**Affiliations:** 1 Geriatric Research, Education and Clinical Center, VA Palo Alto Health Care System, Palo Alto, California, United States of America; 2 Division of Endocrinology, Stanford University, Stanford, California, United States of America; State University of Rio de Janeiro, Biomedical Center, Institute of Biology, BRAZIL

## Abstract

Nordihydroguaiaretic acid (NDGA), the main metabolite of Creosote bush, has been shown to have profound effects on the core components of the metabolic syndrome (MetS), lowering blood glucose, free fatty acids (FFA) and triglyceride (TG) levels in several models of dyslipidemia, as well as improving body weight (obesity), insulin resistance, diabetes and hypertension, and ameliorating hepatic steatosis. In the present study, a high-fructose diet (HFrD) fed rat model of hypertriglyceridemia was employed to further delineate the underlying mechanism by which NDGA exerts its anti-hypertriglyceridemic action. In the HFrD treatment group, NDGA administration by oral gavage decreased plasma levels of TG, glucose, FFA, and insulin, increased hepatic mitochondrial fatty acid oxidation and attenuated hepatic TG accumulation. qRT-PCR measurements indicated that NDGA treatment increased the mRNA expression of key fatty acid transport (L-FABP, CD36), and fatty acid oxidation (ACOX1, CPT-2, and PPARα transcription factor) genes and decreased the gene expression of enzymes involved in lipogenesis (FASN, ACC1, SCD1, L-PK and ChREBP and SREBP-1c transcription factors). Western blot analysis indicated that NDGA administration upregulated hepatic insulin signaling (P-Akt), AMPK activity (P-AMPK), MLYCD, and PPARα protein levels, but decreased SCD1, ACC1 and ACC2 protein content and also inactivated ACC1 activity (increased P-ACC1). These findings suggest that NDGA ameliorates hypertriglyceridemia and hepatic steatosis primarily by interfering with lipogenesis and promoting increased channeling of fatty acids towards their oxidation.

## Introduction

Both obesity and diabetes (~95% type 2 diabetes [T2DM]) have reached epidemic levels in the world [1–3]. Increasing consumption of sugar-sweetened soft drinks, energy dense food, over-nutrition and sedentary lifestyles are the major contributing factors to the obesity and diabetes epidemic. Accompanying obesity is also a constellation of metabolic derangements including insulin resistance, elevated blood pressure and glucose, central obesity, dyslipidemia characterized by low levels of high-density lipoprotein cholesterol and elevated triglycerides, which are commonly referred to as metabolic syndrome (MetS). The explosion of the prevalence of these problems has become a global health concern. MetS increases the risk of cardiovascular disease by approximately 2-fold [2] and raises the risk for T2DM by approximately 5-fold [4]. Nonalcoholic fatty liver disease (NAFLD), the most common cause of chronic liver disease, is strongly associated with core components of MetS and is now considered a hepatic manifestation of the MetS [5–7]. NAFLD encompasses a spectrum of liver diseases from simple steatosis (triglyceride accumulation in the liver) progressing through nonalcoholic steatohepatitis (NASH) and fibrosis to cirrhosis and end-stage liver failure. Like MetS, NAFLD is also a risk factor for T2DM and cardiovascular disease [5–8]. In addition to dysregulated glucose metabolism, all of these metabolic disorders exhibit altered regulation of lipid metabolism, which results in the intracellular accumulation of lipid in nonadipose tissue leading to insulin resistance [6, 9]. Furthermore, hypertriglyceridemia, the most common lipid abnormality, which is characterized by increased production in the liver and/or decreased clearance of triglyceride rich lipoproteins in plasma, contributes to the pathogenesis of these disorders [10, 11]. It is also an independent risk factor for cardiovascular disease [11].

Currently, there are no approved drugs for either the metabolic syndrome or NAFLD, and lifestyle interventions, such as increasing exercise, reducing dietary fat and simple carbohydrate intake, increasing consumption of fiber-rich foods, and losing weight, are the only effective therapeutic strategies with a positive outcome [12–16]. However, this approach has been hampered by a lack of compliance. A second line of pharmacological intervention for MetS and NAFLD includes the use of drugs that are targeted towards improving core components of MetS [14, 16]. But even with the use of combination therapy along with attempted lifestyle management, individual risk factors, particularly dyslipidemia including hypertriglyceridemia in MetS, are often poorly controlled. Moreover, although the use of insulin sensitizers (thiazolidinediones, biguanides), vitamin E (antioxidant), and lipid lowering agents have shown promising results in the management of NAFLD, their therapeutic utility may be limited because of their significant side effects [16].

Creosote bush, *Larrea tridentate*, an evergreen shrub of the southwestern deserts of the United States and Southern Mexico, has been used by Native Americans to treat a variety of ailments including arthritis, diabetes, infertility, gallbladder and kidney stones, pain and inflammation [12]. Previous work from our laboratory [17–19] and others [20, 21] has shown that nordihydroguaiaretic acid (NDGA), the main metabolite of Creosote bush, has profound effects on the core components of the MetS, lowering blood glucose, free fatty acids and triglyceride (TG) levels in several models of dyslipidemia, as well as improving body weight (obesity), insulin resistance, diabetes and hypertension, and ameliorating hepatic steatosis [19].

The present study was initiated to explore the underlying mechanism by which NDGA attenuates hypertriglyceridemia and hepatic steatosis (TG accumulation). These studies were carried out using a unique high fructose diet (HFrD) fed rat model of MetS, manifesting insulin resistance, hypertriglyceridemia, hypertension and hepatic steatosis in the absence of significant weight gain. We provide evidence that NDGA treatment of HFrD fed rats improves hypertriglyceridemia and steatosis by simultaneously targeting (inhibiting) the lipogenic pathway as well as promoting fatty acid channeling towards oxidation in the liver, resulting in a reduction of hepatic VLDL-TG production, storage and secretion.

## Materials and Methods

### Materials

The following reagents were supplied by Sigma-Aldrich Corp. (St Louis, MO): ATP, dithiothreitol (DTT), palmitic acid, fatty acid free bovine serum albumin, deoxycholate, Tris-HCl, SDS, Triton X-100, Oil Red O, and EDTA. Akt (pan) (67E7) rabbit monoclonal antibody (mAb) (#4691), PhosphoPlus^®^Akt (Ser473) (D9E)XP™ rabbit mAb (#4060), phospho-Akt (Thr308) (D25E6) XP® rabbit mAb (#13038), acetyl CoA carboxylase (ACC) rabbit polyclonal antibody (#3662), phospho-acetyl-CoA carboxylase (Ser79) rabbit polyclonal antibody (#3661), AMPKα (23A3) rabbit mAb (#2603), phospho-AMPKα (Thr172) rabbit polyclonal antibody (#2531), AMPKβ1 antibody (#4182), phospho-AMPKβ1 (Ser108) rabbit polyclonal antibody (#4181), phospho-GSK-3α/β (Ser21/9) (37F11) rabbit mAb, and GSK-3β (27C10) rabbit mAb (#9315) were purchased from Cell Signaling Technology (Danvers, MA). MLYCD polyclonal antibody (15265-1-AP) was obtained from Proteintech (Chicago, IL). PPARα (H-98) rabbit polyclonal antibody and SCD1 (E-15) rabbit polyclonal antibody (sc-14720) were obtained from Santa Cruz Biotechnology, Inc. (Santa Cruz, CA). IRDye 800CW-conjugated goat anti-rabbit IgG (H + L) and IRDye 680LT-conjugated goat anti-mouse antibodies were the products of LI-COR Biosciences (Lincoln, NE). Glucose, TG and total cholesterol measurement kits were obtained from Stanbio Laboratory (Boerne, TX), and FFA measurement kits were obtained from Wako Diagnostics. Rat insulin RIA Kit was obtained from EMD Millipore (Billerica, CA). [1-^14^C]-Palmitic acid (40–60 mCi [1.48–2.22 GBq/mmol was purchased from PerkinElmer (Waltham, MA). Humulin^®^R (recombinant human insulin) was the product of Eli Lilly and Company (Indianapolis, IN). NDGA purified from Creosote bush was obtained from Insmed Inc., Richmond, VA as reported earlier [17–19].

### Animals and Treatments

Ethics Statement: All animal experiments were performed according to the procedures approved by the VA Palo Alto Health Care System Institutional Animal Care and Use Committee (IACUC). The specific procedures for this study were approved by the IACUC of VA Palo Alto Health Care System.

Male Sprague-Dawley (SD) rats, obtained from Harlan Laboratory (Indianapolis, IN), were used in these studies. NDGA treatment was delivered to rats weighing 175-200g by oral gavage. Rats were first maintained on a chow diet (TD 2018; Harlan Teklad, Madison, WI) for approximately 1 week to allow them to acclimatize to a controlled new environment (25 ± 2^°^C, 55 ± 5% relative humidity with a 12 h-light-dark cycle), and then divided into three groups, two of which were switched to a high-fructose diet (TD.89247; Harlan Teklad, Madison, WI) that provided 60% of total calories as fructose (HFrD group), while the third group was maintained on a chow diet. Eight weeks (56 days) after feeding, on day 57, the rats were fasted for 4h and tail vein blood was collected for baseline measurement of serum triglyceride (TG), glucose, total cholesterol (TC), insulin and FFA. The two HFrD groups of rats were then treated with vehicle (0.5% carboxymethyl cellulose; CMC) or NDGA (125 mg/kg BW) twice a day for 5 days, delivered by oral gavage. The chow group (diet control) was treated with vehicle. During the treatment regimen, the animals were maintained on the high-fructose diet. After 5 days of treatment, blood was collected from the tail vein 3 h after the last dose of vehicle or NDGA, and serum samples were analyzed for TG, glucose, insulin, TC and FC. Subsequently, rats were euthanized with CO_2_ and liver tissues collected, weighed and quickly frozen in liquid nitrogen. The samples were stored at -80°C for further analysis including Oil Red O staining of frozen sections for steatosis evaluation, RNA extraction for qRT-PCR, lipid extraction for measurement of hepatic triglyceride and cholesterol and protein extraction for Western blot analyses.

### Measurement of Serum Triglyceride, Cholesterol, Glucose, Free-fatty Acids and Insulin Levels

Serum glucose, triglyceride, and total cholesterol levels were determined with commercial assay kits (Stanbio Laboratory, Boerne, TX). Serum free-fatty acids levels were determined with kits from Wako Diagnostic (Richmond, VA). Serum insulin levels were quantified using a rat specific insulin RIA Kit (EMD Millipore, Billerica, CA).

### Quantification of Hepatic Triglyceride Content

Suitable aliquots of liver tissue homogenates were extracted with chloroform-methanol according to the procedure of Folch *et al* [22], and extracted lipid samples were analyzed for their TG content with an enzymatic assay kit as noted above.

### Fatty Acid β-Oxidation Assay

Liver fatty acid (palmitate) oxidation rate was determined in fresh homogenates by a modification of the method of Mannarets *et al* [23] as described previously from this laboratory [19] using [1-^14^C]palmitate. Briefly, approximately 50 to 100 mg of liver samples were homogenized on ice in 20 volumes of SET buffer (250 mM sucrose, 1 mM EDTA, 10 mM Tris-HCl, and 2 mM ATP; pH 7.4) in a Potter-Elvehjem homogenizer with a tight-fitting Teflon pestle. The supernatant fractions in each case were employed for the measurement of total and peroxisomal β-oxidation activities. Briefly, for the determination of total β-oxidation activity, the reactions were carried out in a final volume of 0.4 ml in a 20 ml glass scintillation vial containing 0.2 mM palmitate + 0.2 μCi/ml [1-^14^C]palmitate (complexed to BSA), 100 mM sucrose, 10 mM Tris-HCl, pH 7.4, 10 mM potassium phosphate, 100 mM KCl, 1 mM MgCl_2_, 1 mM L-carnitine, 0.1 mM malic acid, 2 mM ATP, 50 μM Coenzyme A (CoA) and 1 mM DTT. The vial also contained 400 μl of 1N NaOH with filter paper in a well suspended from the top of the vial to trap the ^14^CO_2_ produced during the reaction. Before initiation of the reaction, the vial was sealed with a rubber cap and the reaction was initiated by the addition of 80 μl of the liver homogenate via syringe through the rubber cap. The vial was incubated for 60 min at 30°C before termination with 200 μl 6M H_2_SO_4_. After termination, the flasks were shaken continuously for 2h to ensure quantitative collection of CO_2_ [24]. The paper insert containing the NaOH and trapped ^14^CO_2_ was then removed to a scintillation vial and the radioactivity counted. Peroxisomal β-oxidation assays were carried out under identical conditions as described above except in each case the incubation mixture also contained 2 mM KCN, but without 0.5% BSA. The rate of mitochondrial fatty acid β-oxidation was calculated as the difference of total fatty acid β-oxidation minus peroxisomal fatty acid β-oxidation.

### RNA Isolation and Real Time qRT-PCR

RNA isolation from the liver tissue samples (~20 mg) was carried out using the RNeasy Plus mini kit (Qiagen, Valencia, CA) as per the manufacturer’s instruction and was reverse-transcribed (1 μg total RNA) using a High Capacity RNA-to-cDNA^™^ Kit (Life Technologies, Grand Island, NY). Real-time PCR was performed in a final volume of 10 μl containing 50 ng of cDNA template and specific sets of forward and reverse primers using a FastStart Universal SYBR Green Master PCR Kit (Roche Applied Science, Indianapolis, IN) and an ABI Prism 7700 system (Applied Biosystems^®^ Life Technologies, Grand Island, NY) according to the manufacturer’s protocols. The oligonucleotide primers and their sequences used in RT-PCR are shown in Table 1. The amplification process time was as follows: 95°C for 2 min; 40 cycles at 95°C for 20 s, 60°C for 20 s, 72°C for 30 s, and a final 5-min extension at 72°C. Results were normalized to the housekeeping gene *36B4*, and the values of the control (chow-fed) group were set to 1.

**Table 1:** Primers used in quantitative real-time RT-PCR for detecting gene expression in rat liver tissues.

Gene Name	GenBank Accession Number	Forward Primer (5’ → 3’)	Reverse Primer (5’ → 3’)
*Mlxipl* *	NM_133552.1	TACTGTTCCCTGCCTGCTCT	GTCAGGATGCTGGTGGAAGT
*Srebp-1c*	AF286470	GGCCCTGTGTGTACTGGTCT	AGCATCAGAGGGAGTGAGGA
*Srebp-2*	NM_001033694.1	GGTAATGATGGGCCAAGAGA	GTCCGCCTCTCTCCTTCTTT
*Cebpa* ^¶^	NM_012524.2	GCCAAGAAGTCGGTGGATAA	CGGTCATTGTCACTGGTCAA
*Ppara*	NM_013196.1	TCACACAATGCAATCCGTTT	GGCCTTGACCTTGTTCATGT
*Ppard*	NM_013141.1	TGAGTTCTTGCGCAGTATCC	GCTCCAGAGCATTGAACTTG
*Pparg*	AB011365.1	ACCACGGTTGATTTCTCCAG	CAACCATTGGGTCAGCTCTT
*Foxo1*	XM_342244.3	CCGGAGTTTAACCAGTCCAA	TGCTCATAAAGTCGGTGCTG
*Foxa2*	NM_012743.1	GCTCCCTACGCCAATATGAA	CATGGTGATGAGCGAGATGT
*Xbp1s*	NM_001004210.1	TATCCTTTTGGGCATTCTGG	GAAAGGGAGGCTGGTAAGGAA
*Lxra*	NM_031627.2	TCAGCATCTTCTCTGCAGACCGG	TCATTAGCCATCCGTGGGAACA
*Lxrb*	NM_009473	AAGCAGGTGCCAGGGTTCT	TGCATTCTGTCTCGTGGTTGT
*Fasn*	NM_017332.1	GGATGTCAACAAGCCCAAGT	CAGAGGAGAAGGCCACAAAG
*Acc1*	NM_022193.1	TGAGGAGGACCGCATTTATC	GCATGGAATGGCAGTAAGGT
*Scd1*	NM_139192.2	TGTTCGTCAGCACCTTCTTG	TCTTGTCGTAGGGGCGATAC
*Scd2*	AB032243.1	TCCTGCTCATGTGCTTCATC	GACGCACAGGCTGTTTACAA
*Gpat*	NM_017274.1	GCCATCTTTGTCCACACCTT	CTCTCCGTCCTGGTGAGAAG
*Dgat1*	NM_053437.1	TGCTCTTTTTCACCCAGCTT	TTGAAGGGCTTCATGGAGTT
*Dgat2*	NM_001012345.1	CTTCCTGGTGCTAGGAGTGG	GCCAGCCAGGTGAAGTAGAG
*Plin2*	NM_001007144.1	AATTCGCCAGGAAGAATGTG	CGTAGCCGACGATTCTCTTC
*Mttp*	NM_001007144.1	CCTCCATCCTGATGAAGAA	TCTCTGATGTCGTTGCTTGC
*Arf1*	NM_022518.3	GAAGATGAGCTCCGAGATGC	CCCCAGCTTGTCTGTGATTT
*Pld1*	NM_030992.1	CGAGGAGTTCATCCAGAAGC	TGGCGTTCACACGTACCATTTA
*Fabp1* ^§^	NM_012556.1	AAATCGTGCATGAAGGGAAG	GTCTCCAGTTGGCACTCCTC
*Fabp4* ^†^	NM_053365.1	GAAAGAAGTGGGAGTTGGCT	TACTCTCTGACCGGATGACG
*Fatp5*	NM_024143.2	TCGGATCTGGGAATTCTACG	CAAGCTCAAAGGGAGTCACG
*Cd36*	NM_001109218.1	CCAGAACCCAGACAACCACT	CACAGGCTTTCCTTCTTTGC
*Cpt1a*	NM_031559.2	CAGCTCGCACATTACAAGGA	TGCACAAAGTTGCAGGACTC
*Cpt2*	NM_012930.1	CCAGTATTTCCGGCTTTCA	TTCCCATCTTGATCGAGGAC
*Acox1*	NM_017340.2	CTGATGAAATACGCCCAGGT	GGTCCCATACGTCAGCTTGT
*Lcad*	NM_012819.1	GGCTGGTTAAGTGATCTCGTGAT	TCTCCACCAAAAAGAGGCTAATG
*L-Pk*	NM_012624.3	CTGGATGGGGCTGACTGTAT	GGCGTAGCTCCTCAAACAAC
*Gck*	NM_010292.4	CTATGAAGACCGCCAATGTG	CAGCTCCACATTCTGCATTTT
*Pepck*	NM_198780.3	AATCCGAACGCCATTAAGAC	ATGCCTTCCCAGTAAACACC
*G6pc*	NM_013098.2	ACTCCCAGGACTGGTTTGTC	CCAGATGGGAAAGAGGACAT
*Hsl*	NM_012859.1	ATGGCAGCCTACCCAGTTAC	TTGGAGAGTACGCTCAGTGG
*Cgi-58*	NM_212524.1	CGCATATCCAATGGAAACAG	AACCATGCAGGAGGACAA
*Atgl*	NM_001108509.2	CCTGACTCGAGTTTCGGAT	CACATAGCGCACCCCTTGA
*Lpl*	NM_012598.2	CTCTGTATGGCACAGTGGCT	TCCACCTCCGTGTAAATCAA
*36B4*	NM_007475.5	CAGCAGGTGTTTGACAATGG	CCCTCTAGGAAGCGAGTGTG
*Mlycd*	NM_053477.1	ACTTCTTCTCCCACTGCTCC	GCTTTATGAGGAAGGTGCCG
*Pgc1α*	NM_031347.1	TGTGCAGCCAAGACTCTGTAT	TATGTTCGCGGGCTCATTGT
*Pgc1β*	NM_176075.2	TCTCACACCCCAGTCCAGAA	AGGCTTGTTGACATCCCGTT

*Mlxipl (*Chrebp*);

^¶^ *Cebpa* (*C/ebpa*);

^§^ *Fabp1* (*L-Fabp*);

^†^ *Albp/Ap2* (*Fabp4*)

Abbreviations are: *Acac1*, acetyl-CoA carboxylase 1; *Acox1*, acyl-CoA oxidase 1; *Plin2*, adipose differentiation-related protein; *FABP4*,; *Arf1*, ADP-ribosylation factor 1; *Atgl*, adipose triglyceride lipase; *36B4*, ribosomal protein, large, P0 (RPLP0); *Cebpa*, CCAAT enhancer binding protein alpha; *C/ebpa*, CCAAT enhancer binding protein α; Cd36, cluster of differentiation 36; *Cgi*-58, comparative gene identification-58; *Chrebp*, carbohydrate responsive element-binding protein; *Cpt1a*, carnitine palmitoyltransferase 1A; *Cpt2*, carnitine palmitoyltransferase 2; *Dgat1*, diacylglycerol-O-acyltransferase 1; *Dgat2*, diacylglycerol-*O*-acyltransferase 2; *Fabp1*, fatty acid binding protein 1; *Fabp4*, fatty acid binding protein 4; *Fasn*, fatty acid synthase; *Fatp5*, fatty acid transport protein 5; *Foxo*1, Forkhead box protein O1; *Foxa2*, Forkhead box A2; *G6pc*, glucose-6-phosphatase; *Gck*, glucokinase; *Hsl*, hormone-sensitive lipase; *Lcad*, long-chain acyl-CoA dehydrogenase; *L-pk*, L-type pyruvate kinase; *Lpl*, lipoprotein lipase; *Gpat1*, glycerol-3-phosphate acyltransferase 1; *L-Fabp*, liver fatty acid binding protein; *Lxra*, liver X receptor alpha; *Lxrb*, liver X receptor beta; *Mlxipl*, MLX interacting protein-like; *Mlycd*, malonyl-CoA decarboxylase; *Mttp*, microsomal triglyceride transfer protein; *Ppara*, peroxisome proliferator-activated receptor alpha; *Pepck*, phosphoenolpyruvate carboxykinase; *Pparg*, peroxisome proliferator-activated receptor gamma; *Ppard*, peroxisome proliferator-activated receptor delta; *Pgc1a*, Pparg co-activator 1 alpha; *Pgc1ß*, Pparg co-activator 1 beta; *Pld1*, phospholipase D1; *Scd1*, stearoyl-CoA-desaturase 1; *Scd2*, stearoyl-CoA-desaturase 2; *Srebp-1c*, sterol regulatory element binding protein-1c; *Srebp-2*, sterol regulatory element binding protein-2; *Xbp1s*, X-box binding protein

### Western Blot Analysis

Liver samples (~200 mg) were homogenized using a Potter-Elvehjem homogenizer in three volumes of detergent containing lysis buffer (50 mM Tris.HCl, pH 7.4, 1% Triton X-100 (v/v), 0.1% SDS, 0.25% deoxycholate 150 mM NaCl and 1X Halt™ Protease and Phosphatase Inhibitor Cocktail (Thermo Scientific/Pierce Biotechnology, Rockford, IL) and incubated on ice for 10 min. Following centrifugation at 10,000 x g for 10 min, the supernatant fractions were analyzed for protein content by a BCA™ Protein Assay Kit (Thermo Scientific/Pierce Biotechnology). Nuclear fractions were prepared using NE-PER nuclear and cytoplasmic extraction reagents (Thermo Scientific/Pierce Biotechnology, Rockford, IL). Protein extracts(20 μg) of total or nuclear proteins were subjected to 10% SDS-PAGE under denaturing conditions and transferred to nitrocellulose membranes. Blotted membranes were blocked with Odyssey blocking buffer (LI-COR Biosciences, Lincoln, NE) for 1h and then incubated with anti-phospho Akt, anti-Akt, anti-phospho GSK-3 α/β, anti-GSK-3β, anti-SCD1, anti-PPARα, anti-phospho-AMPKα, anti-phospho-AMPKβ, anti-AMPKβ, anti-phospho ACC1, anti-ACC1, anti-ACC2, or β-actin mAb (Sigma-Aldrich) for 16 h at 4°C. After three washes with tris-buffered saline containing 0.1% Tween 20, the membranes were incubated with IRDye 800CW goat anti-rabbit and IRDye 680LT goat anti-mouse secondary antibodies (LI-COR Biosciences) for 1h. Proteins were detected with the Odyssey Infrared Imaging System (LI-COR Biosciences).

### Statistical Analysis

Statistical analysis was performed using the Prism software (Prism 6.0, GraphPad, San Diego, CA). All of the data are expressed as the means ± S.E. ANOVA analyses were performed with Bonferroni’s multiple comparisons test, *P* < 0.05 was considered statistically significant.

## Results

### NDGA Treatment Improves Plasma Triglyceride (TG), Free-Fatty Acid (FFA) and Insulin Levels in High Fructose Fed Rats

Table 2 compares the effects of feeding a high-fructose diet (HFrD) and oral gavaging of HFrD fed rats with NDGA on body weight, serum glucose, TG, FFA, total cholesterol and insulin concentrations. Body weights were similar among the chow-fed, HFrD and HFrD-NDGA groups, consistent with many previous studies demonstrating that chronic high fructose diets do not cause increases in weight [25]. In contrast, feeding rats the high-fructose diet caused a dramatic elevation in plasma TG levels and also significantly raised plasma FFA and insulin levels. After 4 days of NDGA treatment, NDGA reduced plasma TG levels to chow-fed control levels. NDGA significantly decreased fasting serum insulin, fasting blood glucose and FFA levels. These changes reflected a reversal of insulin resistance induced by fructose feeding by NDGA, as assessed by HOMA-IR. Interestingly, NDGA treatment reduced the FFA levels in HFrD rats to levels that were 40% lower than that of chow-fed control (Table 2).

**Table 2:** Body weight and Plasma glucose, insulin and lipid profiles in Sprague Dawley rats fed a chow diet (n = 4), a high-fructose diet (HFrD, n = 6) or a HFrD and treated with NDGA (n = 6).

**Parameter**	**Chow-Vehi (Control)**	**HFrD-Vehi**	**HFrD-NDGA**
Total body weight (g)	387.50 ± 14.16	372.30 ± 8.42	386.50 ± 19.67
Plasma Triglyceride (mg/dl)	94.50 ± 13.69	348.50 ± 17.61***	84.33 ± 12.72
Plasma cholesterol (mg/dl)	121.50 ± 7.67	111.30 ± 6.63	111.00 ± 4.66
Plasma glucose (mg/dl)	135.30 ± 14.67	138.70 ± 2.73	124.70 ± 4.78†
Plasma free fatty acids (mEq/l)	0.64± 0.10	0.81 ± 0.25	0.38 ± 0.03* †
Plasma insulin (ng/ml)	0.47 ± 0.04	0.73 ± 0.05**	0.67 ± 0.04**
HOMA-IR	3.75±0.0003	6.00±0.0001***	4.95±0.0001*** ††

Body weight and blood chemistry were assayed as described in material and methods, data are presented as means ± SE.

* *P*<0.05;

** *P<0*.*01*;

*** P<0.*001* compared to chow.

^†^ P<0.05,

^††^ P<0.01 compared to HFrD. Raw data and statistics are listed in S1 Dataset.

### NDGA Treatment Alleviates High Fructose Feeding Induced Hepatic TG Accumulation

**Fig 1:**
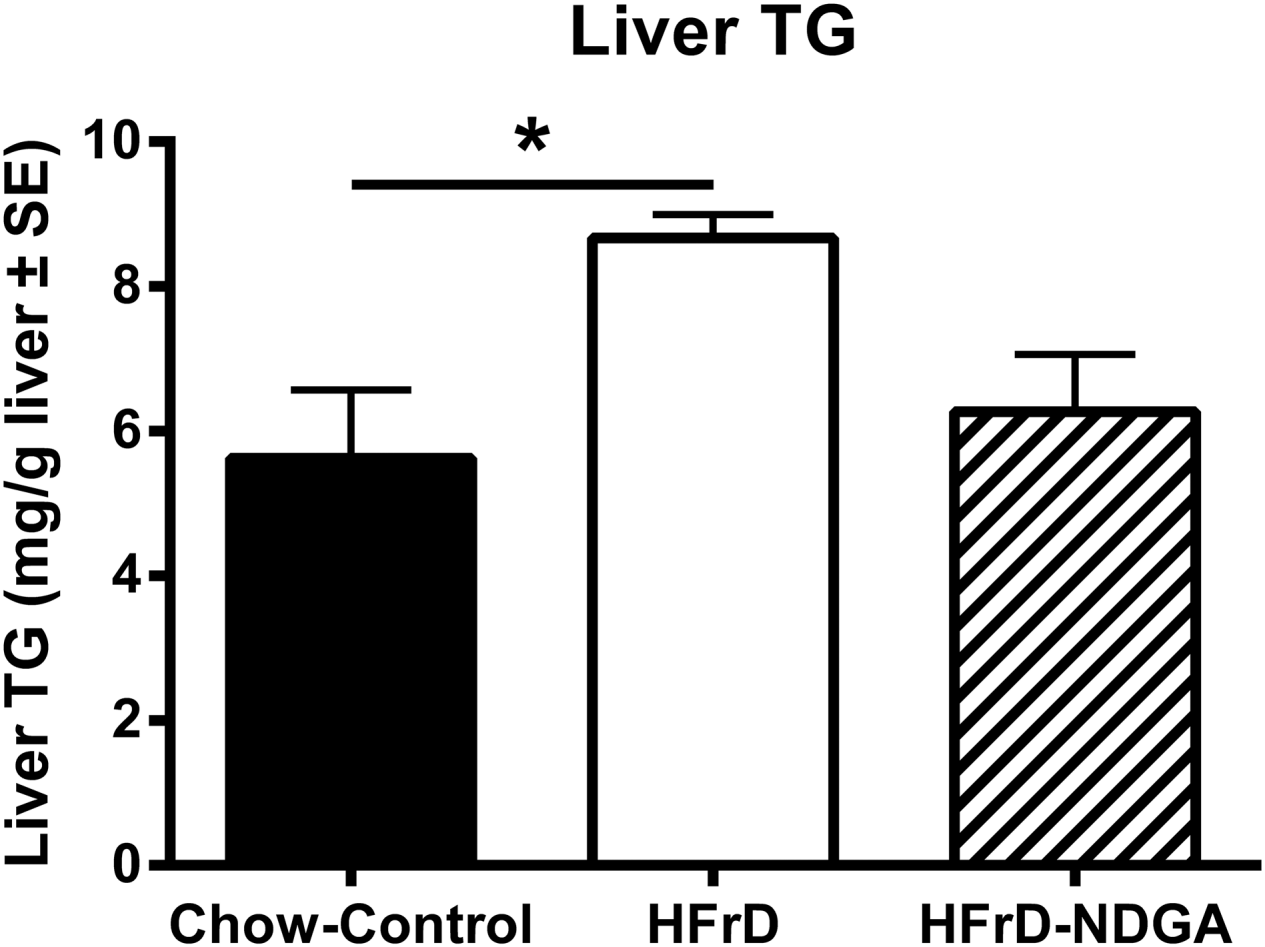
Effect of orally administered NDGA on liver weights (A) and triglyceride content (B) in high-fructose fed (HFrD) hypertriglyceridemic rats. Groups of rats were maintained on a chow-diet (n = 4) or HFrD (n = 12) for 8 weeks and, subsequently, HFrD fed rats were divided into two groups: one was orally gavaged with NDGA (n = 6) at a dose of 125 mg/kg BW twice a day for 5 days and the other group received vehicle (n = 6) (control). Liver samples were subsequently weighed (A) and quantified for TG content (B), as described in the Materials and Methods. Values are mean ± SE. * *P*<0.05; ** *P<0*.*01*; *** *P<0*.*001*. Raw data and statistics are listed in S1 Dataset.

The results presented in Fig 1A show that fructose feeding increased hepatic weights, which was unaffected by NDGA treatment. Hepatic TG content of HFrD animals was elevated compared with chow controls (*P* < 0.05). NDGA treatment showed a trend in reducing hepatic TG (Fig 1B and 1C), an effect consistent with the restoration of normal plasma TG levels (Table 2).

### NDGA Treatment Increases Hepatic Fatty Acid β-Oxidation

**Fig 2:**
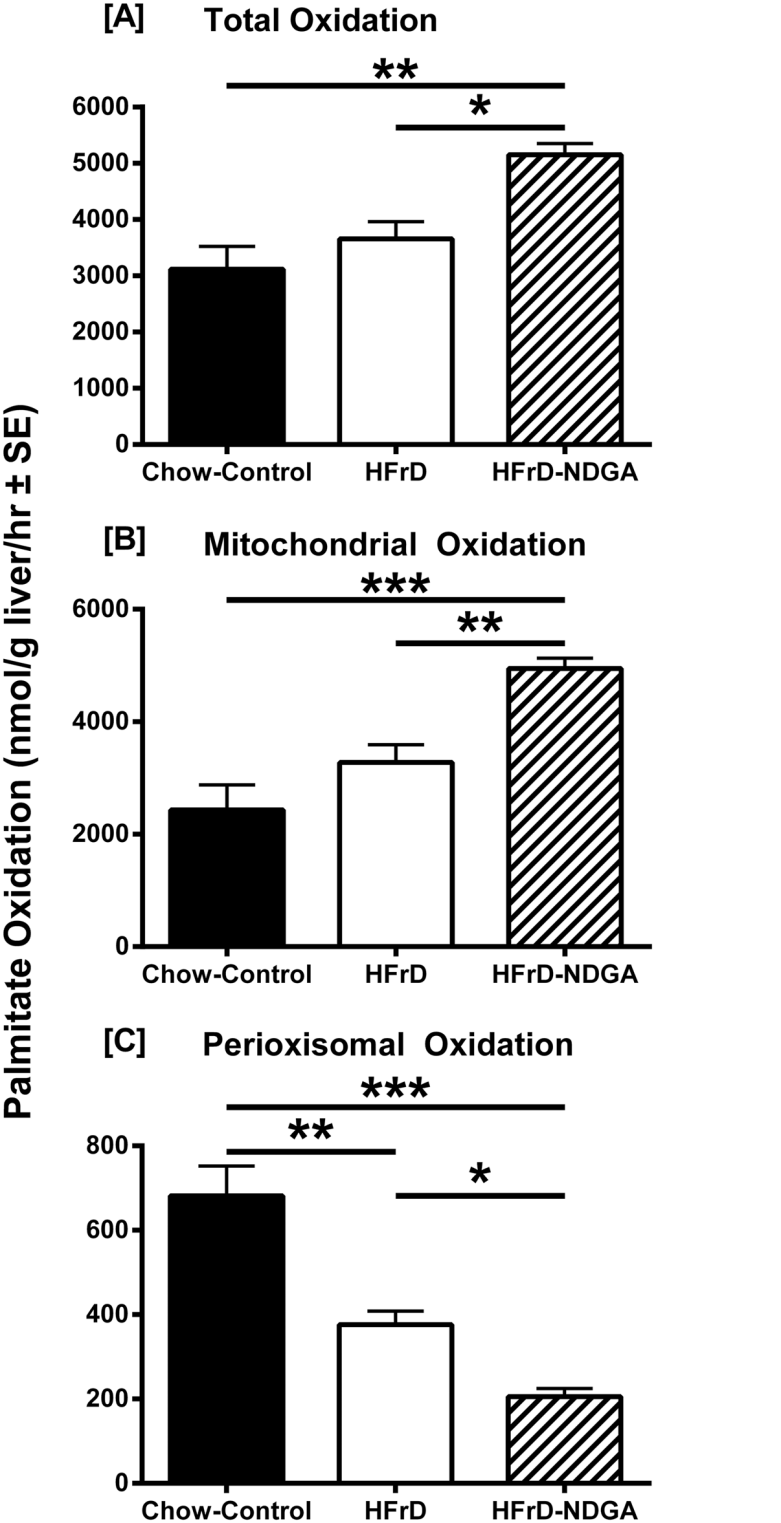
Effect of oral administration of NDGA on hepatic fatty acid oxidation in high-fructose diet (HFrD) fed hypertriglyceridemic rats. Groups of rats were maintained on a chow-diet or HFrD for 8 weeks and, subsequently, HFrD were divided into two groups: one was orally gavaged with NDGA at a dose of 125 mg/kg BW twice a day for 5 days and the other group received vehicle (control). Freshly excised liver samples were processed for the measurement of total, mitochondrial and peroxisomal fatty acid oxidation rates. Values are mean ± SE. * *P*<0.05; ** *P<0*.01; *** P<0.*001*. Raw data and statistics are listed in S1 Dataset.

In an effort to investigate the underlying mechanisms involved in NDGA-mediated amelioration of hypertriglyceridemia, we first measured hepatic mitochondrial and peroxisomal fatty acid β-oxidation rates in liver samples from chow-fed, HFrD-fed and HFrD-NDGA-treated animals. The results presented in Fig 2 demonstrate that hepatic total and mitochondrial β-oxidation ^14^CO_2_ production rates were similar between HFrD fed and chow-fed control groups. Feeding the high-fructose diet, however, significantly decreased the rate of hepatic peroxisomal dependent β-oxidation production of ^14^CO_2_ as compared to data obtained with chow-fed control livers (*P* < 0.01). Treatment of animals with NDGA further decreased the rate of peroxisomal fatty acid oxidation (*P* <0.05 HFrD vs HFrD-NDGA; P <0.0001 Chow vs HFrD-NDGA). In contrast, total and mitochondrial fatty acid oxidation was 40–50% greater in HFrD-NDGA-treated animals compared to vehicle administered HFrD-fed rats (*P* <0.05 total HFrD vs total HFrD-NDGA; *P* <0.01 Mito HFrD vs Mito HFrD-NDGA).

### NDGA Treatment Decreases Expression of Genes Involved in Hepatic Lipogenesis and Increases Expression of Genes Involved in Hepatic Fatty Acid β-Oxidation

**Fig 3:**
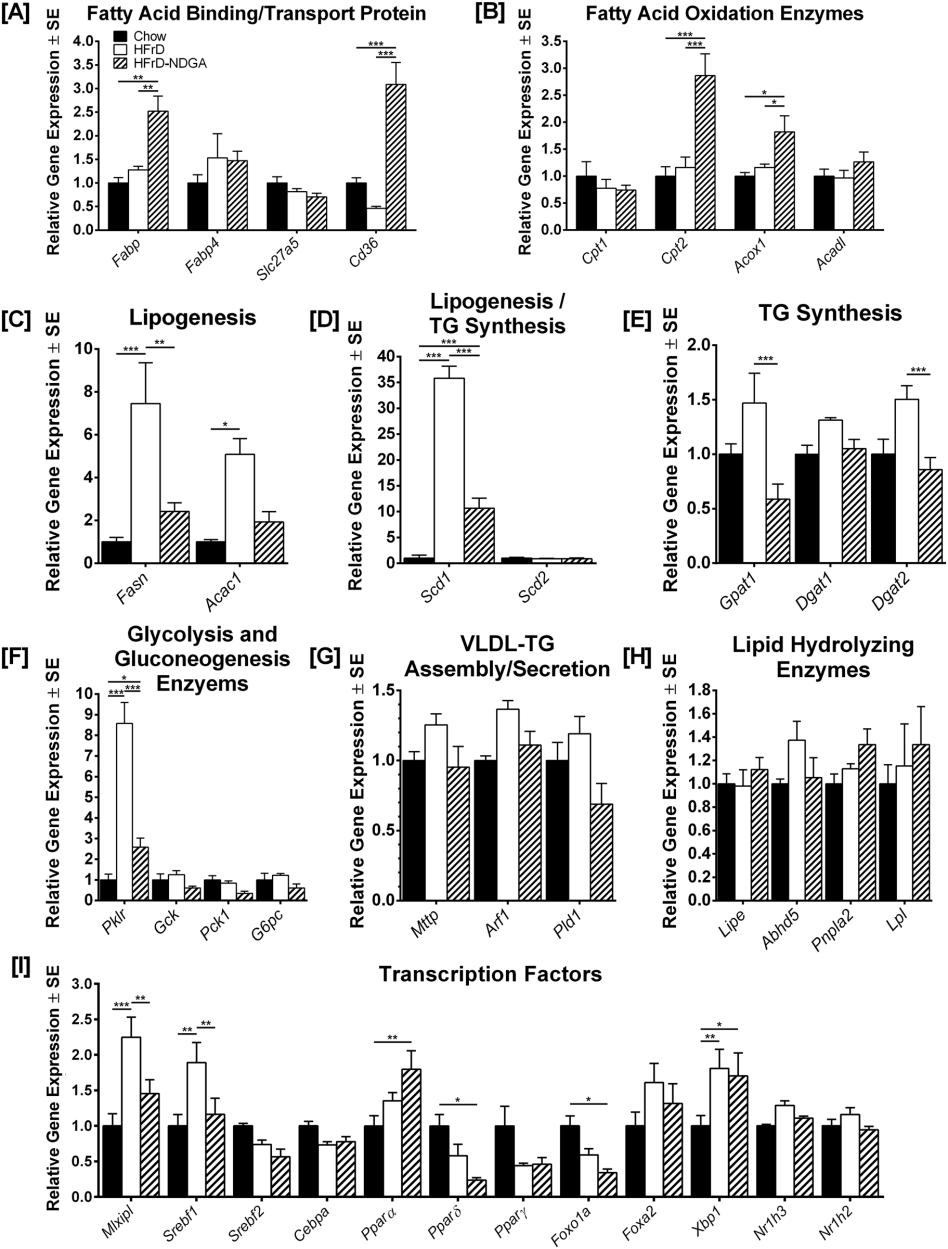
Effect of acute NDGA treatment on the expression of genes and transcription factors involved in hepatic fatty acid metabolism and development of hepatic steatosis in high-fructose diet (HFrD) fed hypertriglyceridemic rats. Quantitative RT-PCR of RNA from livers of chow-fed rats, rats fed an HFrD for 8 weeks and rats fed an HFrD for 8 weeks and subsequently treated with NDGA at a dose of 125 mg/kg BW twice a day by oral gavage. Results are mean ± SE of 3 independent RT-PCR experiments for each gene with 6 rats within each group. Data are presented relative to *36B4* in the same samples, and relative mRNA levels were determined by qRT-PCR using the comparative C_T_ method. * *P*<0.05; ** *P<0*.01; *** P<0.*001*. Raw data and statistics are listed in S1 Dataset.

To better understand the NDGA mediated changes in plasma and liver triglyceride levels and hepatic fatty acid β-oxidation rates, mRNA levels of key proteins and lipogenic transcription regulators involved in hepatic lipid metabolism were quantified by real-time reverse-transcriptase polymerase chain reaction (qRT-PCR). The results are summarized in Fig 3. The mRNA levels of two key fatty acid binding/transport proteins, *Fabp* (L-FABP) and *Cd36* (CD36/FAT), were approximately 2.5-fold higher in liver samples from HFrD-NDGA treated rats as compared to rats fed HFrD alone (Fig 3A; *P* <0.01 HFrD vs HFrD-NDGA and *P* <0.0001 HFrD vs HFrD-NDGA, respectively). In contrast, no changes were noted in the expression levels of *Fabp4* (FABP4) and *Slc27a5* (FATP5) in the HFrD-NDGA group compared to the HFrD or chow control group. With regard to fatty acid oxidation, we observed a significant increase in *Acox1* expression in HFrD-NDGA treated rats (Fig 3B; *P*< 0.05HFrD-NDGA vs HFrD); the protein encoded by this gene, acyl-CoA oxidase (ACO), is the first enzyme in the fatty acid β-oxidation pathway and catalyzes the desaturation of acyl-CoAs to 2-trans-enoyl-CoAs, and is the rate-limiting step in peroxisomal fatty acid β-oxidation [26]. Expression of *Cpt2*, which encodes carnitine palmitoyl-transferase II (CPT-II), another enzyme that participates in fatty acid oxidation, was also upregulated by HFrD-NDGA treatment (Fig 3B; *P* <0.0001 HFrD-NDGA vs HFrD). The expression of other mitochondrial β-oxidation enzymes, such as Cpt1 (CPT-1) and Acadl (acyl CoA dehydrogenase, long chain), was not impacted by either HFrD feeding or HFrD-NDGA treatment.

**Fig 4:**
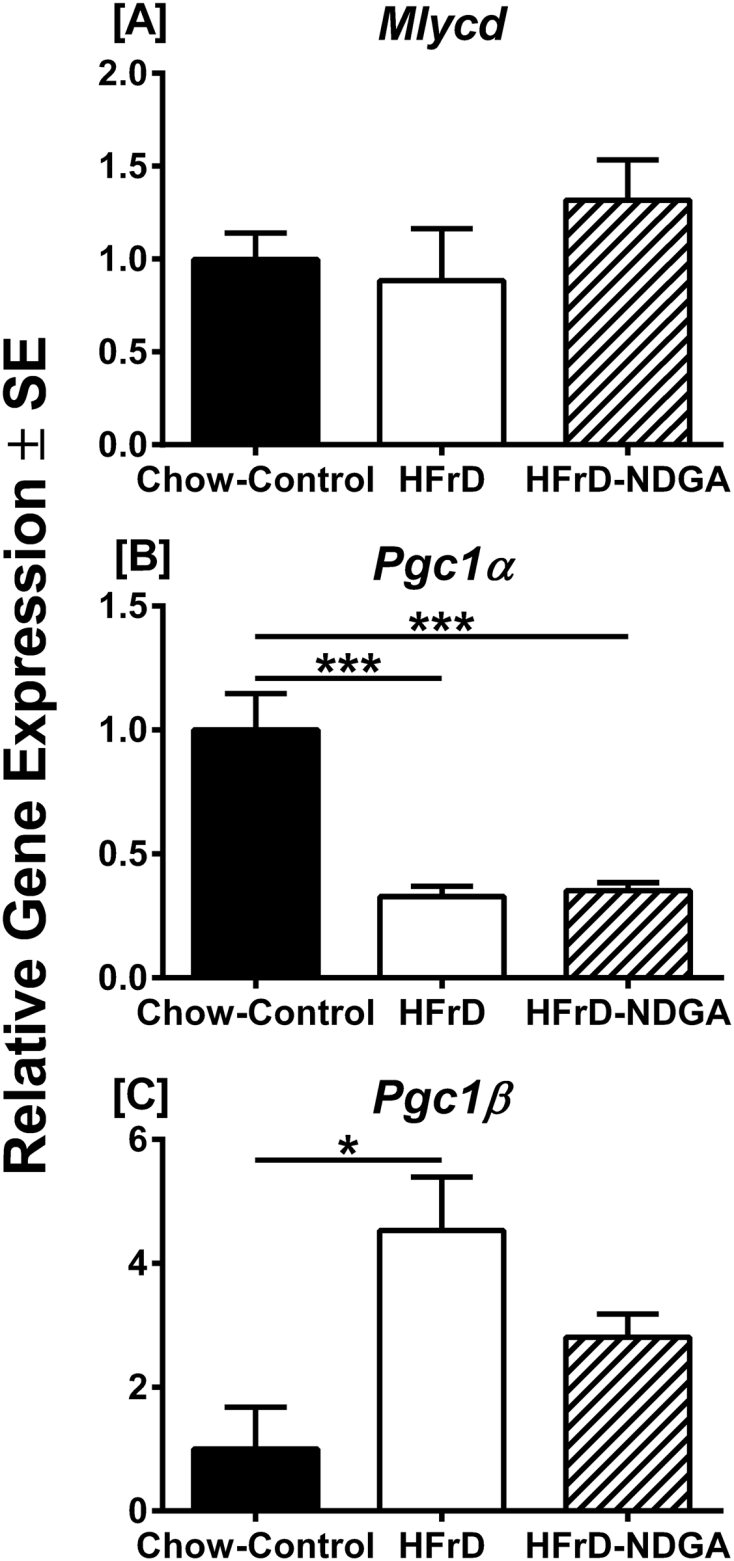
Effect of NDGA treatment on expression of Mlycd, Pgc-1α and Pgc-1β. Quantitative RT-PCR of RNA from livers of chow-fed rats, rats fed an HFrD for 8 weeks and rats fed an HFrD for 8 weeks and subsequently treated with NDGA at a dose of 125 mg/kg BW twice a day by oral gavage. Results are mean ± SE of 3 independent RT-PCR experiments for each gene within each group (normal chow, n = 3, high fructose n = 4, high fructose with NDGA n = 4). Data are presented relative to *36B4* in the same samples, and relative mRNA levels were determined by qRT-PCR using the comparative C_T_ method. * *P*<0.05; ** *P<0*.01; *** P<0.*001*. Raw data and statistics are listed in S1 Dataset.

We measured the expression of three genes involved in fatty acid synthesis (*Acac1*, *Fasn*, and *MLYCD*). *Acac1* encodes acetyl CoA carboxylase, which catalyzes irreversible carboxylation of acetyl CoA to produce malonyl CoA. The *Fasn* gene product, fatty acid synthase complex, catalyzes the synthesis of palmitate from acetyl CoA and malonyl CoA [26, 27], whereas *MLYCD* encodes malonyl CoA decarboxylase, which catalyzes malonyl CoA degradation [28]. The expression levels of *Acac1* and *Fasn* mRNA were up-regulated in response to high-fructose feeding (*P* <0.05 Chow vs HFrD and *P* <0.0001 Chow vs HFrD, respectively), but down-regulated with NDGA treatment (Fig 3C; *P* <0.05 HFrD vs HFrD-NDGA and *P* <0.001 HFrD vs HFrD-NDGA, respectively). In contrast, the expression levels of MLYCD mRNA were unaffected by either the HFrD or NDGA treatment (Fig 4). Moreover, the fatty acid desaturation enzyme, *Scd1* (SCD1), but not *Scd2* (SCD2), which participates in TG synthesis showed similar expression patterns to the fatty acid synthesis enzymes except high-fructose feeding greatly upregulated (~9-fold) *Scd1* mRNA levels (Fig 3D). Likewise, mRNA expression of other TG synthesizing enzymes, *Gpat1* and *Dgat2*, was up-regulated in response to HFrD feeding, whereas oral administration of NDGA reduced their expression to chow fed control levels (Fig 3E). Similarly, the expression of *Pklr* (L-PK), which also contributes to lipogenesis, was up-regulated in HFrD animals (*P* <0.0001 Chow vs HFrD) and reduced to normal levels following NDGA treatment (Fig 3F, *P* <0.001, HFrD vs HFrD-NDGA). In contrast, the expression levels of key genes involved in gluconeogenesis (*Gck*, *Pck1* and *G6pc*) were not altered in HFrD or HFrD-NDGA groups (Fig 3F). We examined the expression of genes that participate in VLDL-TG assembly/secretion (*Mttp* [MTP], *Arf1* [ARF1], and *Pld1* [PLD1]) or lipid hydrolyzing enzymes, *Lipe* [HSL], *Abhd5* [CGI-58], *Pnpla2* [ATGL] and *Lpl* [LPL]), all of which showed comparable mRNA expression levels among the three groups (Fig 3G and 3H).

We next evaluated the effect of HFrD and HFrD-NDGA treatment on several key hepatic lipogenic transcription regulators. Among these, the carbohydrate response element-binding protein (ChREBP, also known as MLX-interacting protein-like) and sterol regulatory element binding protein-1c (SREBP-1c) are the two major transcription factors that induce key lipogenic enzymes to promote lipogenesis and steatosis [29–32]; ChREBP also increases the expression of a glycolytic gene, *Pklr*, and its protein product, L-PK, which in turn, stimulates both glycolysis and lipogenesis [30, 32]. SREBP-1c is activated by insulin via both transcriptional and posttranscriptional mechanisms [32, 33], whereas ChREBP is activated by high glucose independently of insulin [30, 32]. Feeding a high-fructose diet increased both *Mixipl* (ChREBP) and *Srebf1* (SREBP-1c) mRNA levels by approximately 2-fold (*P* <0.001 and *P* <0.01 vs Chow, respectively) (Fig 3I). In HFrD-NDGA livers, however, mRNA levels of both *Mixipl* and *Srebf1* were reduced (*P* <0.01 and *P* <0.01 vs HFrD, respectively). The mRNA levels of *Ppara* (PPARα), a nuclear receptor and master regulator of the fatty acid oxidation pathway in the liver [34, 35], was increased in HFrD-NDGA treated (*P* <0.01) rats as compared to animals maintained on a chow diet (Fig 3I), but high-fructose feeding had no effect. In contrast, the expression of *Ppard* (PPARδ), which also participates in the regulation of energy metabolism, was decreased by 70–80% in response to HFrD-NDGA treatment as compared to the chow-fed group. The expression of nuclear receptor *Pparg* (PPARγ), which promotes lipogenesis, as well as Cebpa (C/EBPα), *Nr1h2* (LXRβ) and *Nr1h3* (LXRα), was not affected by either of the treatments (Fig 3I). The expression levels of *Foxo1a* (FOXO1A) and *Foxa2* (FOXA2) were down-regulated and up-regulated by HFrD-NDGA treatment (*P* <0.05) and HFrD feeding (*P* <0.05), respectively. Similarly, Xbp1 (XBP-1) mRNA levels were up-regulated both in high-fructose fed and HFrD-NDGA treated groups. PGC1α mRNA expression was down-regulated by fructose feeding and unaffected by NDGA, whereas levels of PGC1ß mRNA were elevated by fructose, but was not changed by NDGA treatment (Fig 4). The mRNA levels of malonyl-CoA decarboxylase (MLYCD) were not impacted by either fructose feeding or NDGA treatment (Fig 4).

### NDGA Treatment Stimulates the Phosphorylation of AMPK, ACC, AKT and GSK3, as well as PPARα and malonyl-CoA decarboxylase Protein Levels, and Suppresses SCD1 and ACC Protein Expression

**Fig 5:**
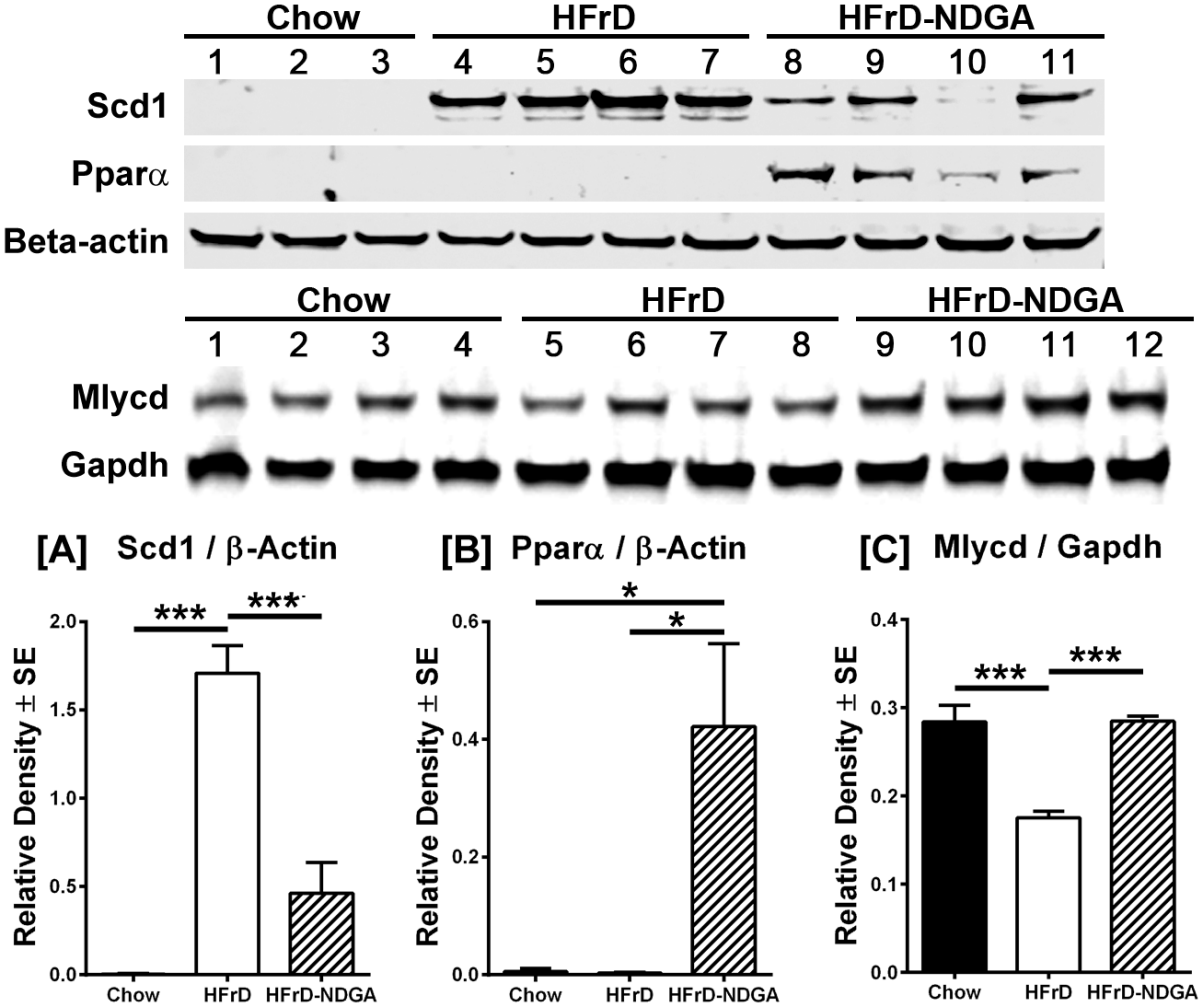
Effect of NDGA treatment by oral gavage on protein expression of SCD1, PPARα and Mlycd protein. After 8 weeks of chow, HFrD feeding or HFrD feeding-NDGA treatment, animals were fasted for 4h before tissue collection and liver homogenates were immunoblotted for SCD1, PPARα and Mlycd protein. Representative blots for these proteins are shown. Data presented are means ± SE of 3–4 individual samples. * *P*<0.05; ** *P<0*.01; *** P<0.*001*. Raw data and statistics are listed in S1 Dataset.

**Fig 6:**
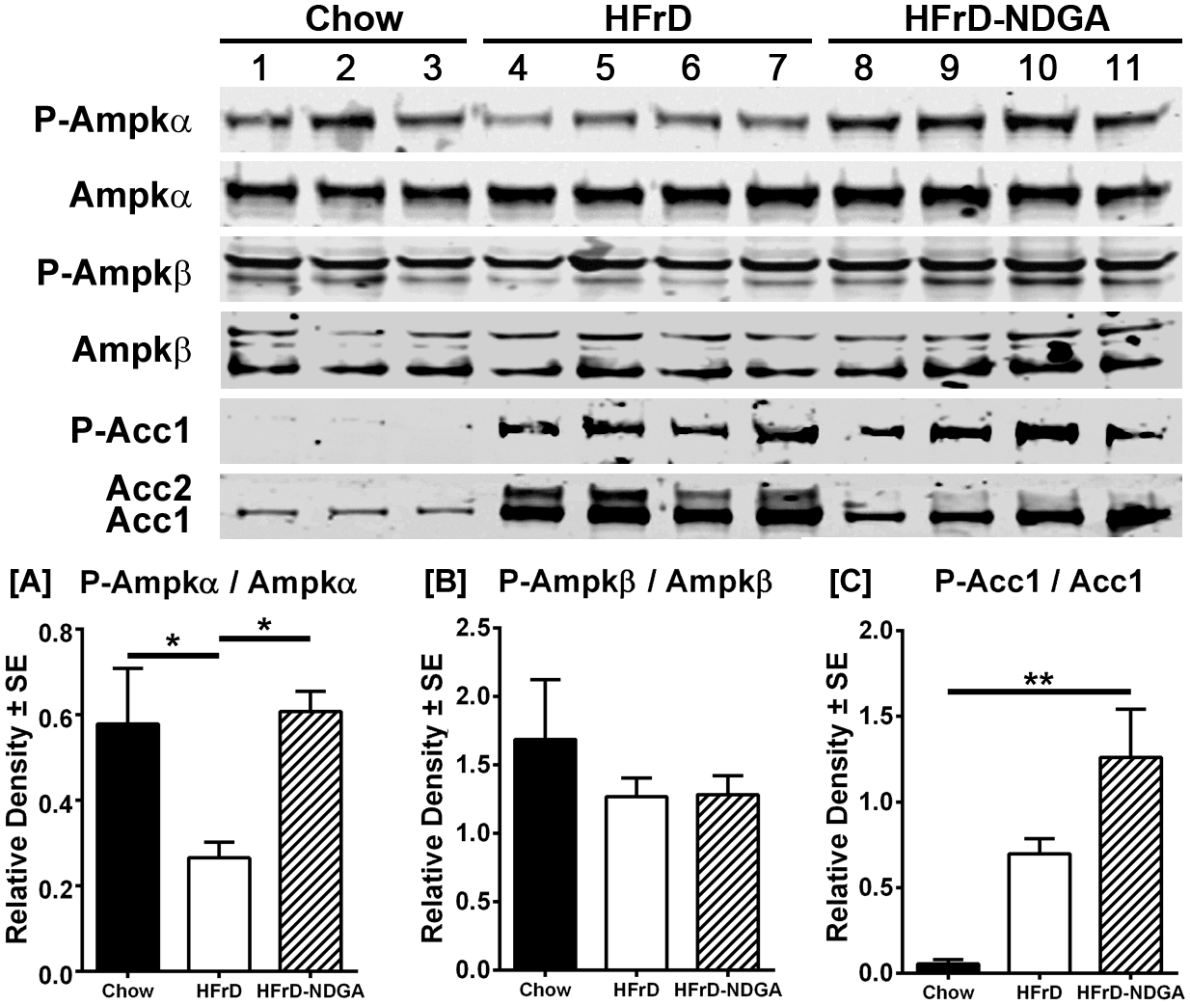
Effect of NDGA treatment by oral gavage on expression and phosphorylation of AMPKα, AMPKβ, ACC1 and ACC2. After 8 weeks of chow, HFrD feeding or HFrD feeding-NDGA treatment, animals were fasted for 4h before tissue collection and liver homogenates were immunoblotted for P-AMPKα, total AMPKα, P-AMPKβ, total AMPKβ, P-ACC1 and total ACC1 and ACC2. Representative blots for these proteins are shown. Data presented are means ± SE of 3–4 individual samples. * *P*<0.05; ** *P<0*.01; *** P<0.*001*. Raw data and statistics are listed in S1 Dataset.

The data presented above indicate that NDGA treatment stimulates and inhibits the gene expression of key enzymes involved in fatty acid oxidation and lipogenesis, respectively. Here, we measured the protein levels of two lipogenic enzymes, ACC and SCD1, and the PPARα, the master regulator of fatty acid oxidation together with malonyl-CoA decarboxylase. As shown in Fig 5, NDGA treatment suppressed the fructose-induced increases in SCD1 protein. Hepatic PPARα protein level was almost undetectable in HFrD fed mice, butwas increased significantly by NDGA treatment. At the same time, NDGA prevented the fructose suppression of MLYCD (Fig 5). In addition, we measured the total and phosphorylated levels of AMPK, ACC, AKT, and GSK3α/β. AMPK, a heterotrimeric protein complex consisting of a catalytic α subunit and regulatory β and γ subunits, plays a key role in the regulation of energy homeostasis [36, 37]. Phosphorylation of Thr172 within the α subunit and Ser108 of the β1 subunit by upstream kinases results in the activation of AMPK activity [36, 37]. AMPK phosphorylates ACC1 on Ser79 (the major phosphorylation site), Ser1200 and Ser1215 residues and deactivates its activity [38]. AKT, a core component of the phosphoinositol 3-kinase (PI3K)-AKT/protein kinase B (PKB), which is responsible for most of the metabolic actions of insulin [39], is activated by phosphorylation at Ser473 and Thr308. GSK3 is rapidly phosphorylated at Ser21 in GSK3α or Ser9 in GSK3β by AKT, resulting in inhibition of GSK3 kinase activity [40]. Results presented in Figs 5 and 6 demonstrate that protein levels of ACC1, ACC2 and SCD1, similar to their mRNA levels, were up-regulated in livers of high-fructose fed rats as compared to chow-fed control livers. Treatment of HFrD fed animals with NDGA decreased the protein levels by approximately 75–80%, further confirming that NDGA can inhibit hepatic lipogenesis. In contrast, the protein expression of PPARα was barely detectable in control or HFrD livers, but was markedly induced in response to HFrD-NDGA feeding (Fig 5).

**Fig 7:**
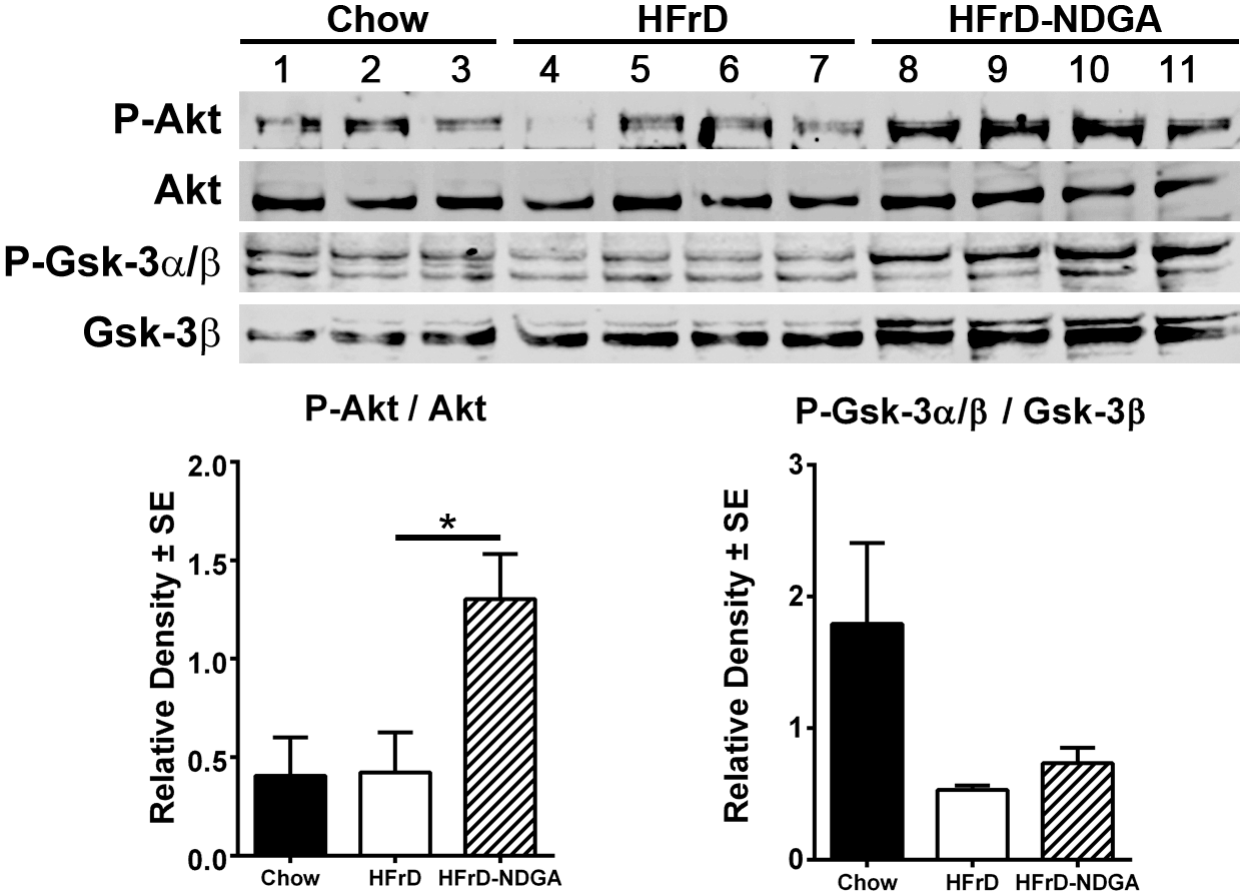
Effect of NDGA treatment by oral gavage on expression and phosphorylation of Akt and GSK3β. After 8 weeks of Chow, HFrD feeding or HFrD feeding-NDGA treatment, animals were fasted for 4h before tissue collection and liver homogenates were immunoblotted for P-Akt, total Akt, P-GSKα/β, and total GSK3β. Representative blots for these proteins are shown. Data presented are means ± SE of 3–4 individual samples. * *P*<0.05; ** *P<0*.01; *** P<0.*001*. Raw data and statistics are listed in S1 Dataset.

Additionally, Western blotting analysis demonstrated that HFrD feeding decreased, whereas HFrD-NDGA treatment restored, the levels of the phosphorylated form of AMPKα (P-AMPKα) (Fig 6). Protein expression of hepatic total AMPKα, P-AMPKβ and total AMPKβ, however, was similar between chow-fed, HFrD and HFrD-NDGA animals (Fig 6). Interestingly, ACC1 phosphorylation was markedly increased by HFrD feeding, which was further increased in response to NDGA treatment (Fig 6; *P* <0.05). Total AKT levels were comparable among the three groups, but NDGA treatment significantly increased the basal Ser473 phosphorylation of AKT by approximately 2.5-fold (Fig 7). Although basal phosphorylation of both GSK3α (Ser21) and GSK3β (Ser9) tend to be increased, this difference was not statistically significant when data were normalized to total GSK3β (Fig 7). Moreover, total GSK3β levels showed a trend to be increased in response to both HFrD and HFrD-NDGA compared to chow fed animals; however, this did not achieve statistical significance.

## Discussions

The objective of the present study was to examine the underlying mechanisms by which NDGA attenuates hypertriglyceridemia and hepatic steatosis in a novel high-fructose diet fed rat model not associated with excessive weight gain. Specifically, we used qRT-PCR to identify lipid genes and pathways and modulators of lipid metabolism that might be the targets of NDGA amelioration of hypertriglyceridemia and steatosis. We examined gene expression in liver, and identified a number of genes involved in fatty acid oxidation, lipogenesis, and lipid clearance, as well as transcription factors influencing lipid metabolism, that were significantly up-regulated or down-regulated in response to NDGA treatment. In addition, Western blot measurements indicated that NDGA mediated the activation of liver AMPK, a core regulator of cellular energy homeostasis, as well as upregulated hepatic insulin signaling [39–42].

Our metabolic measurements indicated that oral gavage of HFrD fed rats with NDGA significantly decreased plasma glucose, fatty acids, TG and insulin levels nearly to levels observed in chow fed control, and attenuated hepatic steatosis as assessed by determination of liver TG content. The decrease in hepatic steatosis as a result of NDGA treatment may help alleviate insulin resistance. Indeed, NDGA treatment significantly upregulated hepatic insulin signaling, as evidenced by an improvement in insulin resistance. Hepatic and muscle insulin resistance is a common pathological feature in human patients with type 2 diabetes. A potential mechanism by which steatosis (ectopic lipid accumulation) induces insulin resistance is due to an accumulation of diacylglycerol (DAG), a precursor and hydrolysis product of TG, which activates protein kinase C and thereby causes impaired insulin signaling and insulin resistance in liver and skeletal muscle [9, 43]. Ceramides have also been implicated in the pathogenesis of insulin resistance. Ceramide levels have been shown to be elevated in plasma and skeletal muscle of patients with type 2 diabetes and in liver, plasma and muscle of obese mice[44, 45]. Whether, hepatic ceramides contribute to insulin resistance remains controversial. For example in some animal models with enhanced steatosis and elevated ceramide levels, insulin sensitivity was not affected [46, 47], and also in obese subjects, no correlation was observed between hepatic ceramides and hepatic insulin resistance [48]. Likewise, recently it has been shown that saturated fat-induced hepatic insulin resistance is independent of ceramides [49]. In contrast, several other studies have reported a significant association between hepatic ceramide concentrations and insulin resistance [45, 50–52]. Another prevailing view is that UPR-mediated activation of the Jun-N-terminal kinase (JNK) leads to serine307 phosphorylation of insulin receptor substrate-1 (IRS-1), which in turn leads to impaired insulin signaling and insulin resistance. As NDGA has been reported to inhibit protein kinase C [53] and JNK [18, 54] activities, it is likely that the NDGA attenuation of hepatic insulin resistance observed in the current studies involves inhibition of one or both of these kinases. Indeed, we found that NDGA treatment leads to upregulation of hepatic mitochondrial fatty acid oxidation and a number of FA oxidation genes in liver. Thus, increased fatty acid oxidation induced by NDGA may be a major contributing factor in reducing lipid accumulation and consequently insulin resistance.

The qRT-PCR measurements indicated that HFrD feeding promoted hepatic TG accumulation (hepatic steatosis) along with increased expression of lipogenic genes that contribute to hepatic steatosis such as *Fasn*, *Acac1*, *Scd1*, *Gpat1 and Dgat2*. Furthermore oral gavaging HFrD rats with NDGA caused a significant reduction in lipogenic transcription factors, *Srebf1* and *Mlxipl*, and their down-stream target genes (*Fasn*, *Acac1*, *Scd1*, and *Pklr*), which participate in *de novo* lipogenesis. Although *Srebf1* and *Mlxipl* are also regulated post-transcriptionally, these data demonstrate that one of the beneficial anti-lipidemic actions of NDGA involves the inhibition of hepatic lipogenesis. Our data also show that expression of some of the genes involved in hepatic fatty acid uptake (*Fabp* and *Cd36*) and oxidation (*Cpt2*, and *Acox1*) and *Ppara*, was variably upregulated in response to NDGA treatment. PPARα is a key transcription factor of fatty acid oxidation that regulates peroxisomal and mitochondrial β-oxidation and microsomal ω-oxidation of FA, with the liver being a major site of action [34, 35, 55]. In agreement with a previous report [19], NDGA treatment was effective in stimulating PPARα expression as demonstrated by increased expression of both its mRNA and protein levels. In line with the positive regulation of PPARα expression, NDGA enhanced mitochondrial β-oxidation; however, it should be noted that NDGA did not upregulate all PPARα-regulated genes for currently unclear reasons. More importantly, NDGA enhanced the expression of target genes, Fabp and Cd36 [35, 55], whose protein products (L-FABP and CD36, respectively) facilitate uptake and transport of fatty acids [56]. Thus, NDGA not only upregulates PPARα and promotes mitochondrial β-oxidation, but also increases the availability of fatty acids for their oxidation by up-regulating the expression of two major fatty acid transport proteins. mRNA expression of most of the genes involved in hepatic TG synthesis, VLDL-TG assembly, lipid clearance and other lipogenic transcription factors except *Dogat1*, *Dgat2*, *Arf1*, *Ppard* and *Foxo1a*, however, were not altered by acute treatment of HFrD rats with NDGA.

We further observed that NDGA treatment increased the phosphorylation (activation) of AMPK, a core regulator of cellular energy homeostasis [36, 37] and the levels of phospho-ACC1. One of the major actions of AMPK is to inhibit lipogenesis and to promote fatty acid oxidation by altering cellular levels of malonyl-CoA [57]. Liver AMPK decreases hepatic lipogenesis by phosphorylating and inactivating ACC1 and also by suppressing the expression of lipogenic enzymes (FASN and ACC) via decreasing the actions of lipogenic transcription factors SREBP-1c [58] and ChREBP [59]. Given this, it is highly likely that NDGA inhibits hepatic lipogenesis, not only by directly inhibiting the transcription of lipogenic genes, but also by interfering with the functions of SREBP-1c and ChREBP via AMPK, leading to suppression of FASN and ACC gene transcription and ACC catalytic activity.

Malonyl-CoA is both a critical substrate for fatty acid biosynthesis and also serves as an inhibitor of carnitine palmitoyltransferase 1 (CPT-1), the rate-limiting enzyme that facilitates the transport of FAs from their cytosolic compartment to the mitochondria for oxidation [60, 61], and, thus, functions as a potent inhibitor of fatty acid oxidation [28, 61]. Previous studies have shown that AMPK reduces malonyl-CoA levels by a dual mechanism: inhibition of ACC activity by AMPK-mediated increased phosphorylation of the protein [62] and AMPK-dependent activation of malonyl-CoA decarboxylase activity [63], which catalyzes malonyl CoA degradation [28], resulting in enhanced fatty acid oxidation. Although we did not directly measure malonyl-CoA levels, our demonstration of NDGA-mediated increased ACC1 phosphorylation (inactivation of activity) strongly suggests the possibility that in addition to directly upregulating the mitochondrial fatty acid β-oxidation pathway and FA transport proteins, NDGA may also promote FA oxidation by decreasing the cellular levels of malonyl-CoA through AMPK-mediated inhibition of ACC and activation of malonyl-CoA decarboxylase. Although hepatic Mlycd mRNA levels, the gene encoding malonyl-CoA decarboxylase, did not change significantly, we noted a significant induction of malonyl-CoA decarboxylase protein levels in response to NDGA treatment; malonyl-CoA decarboxylase is transcriptionally regulated by PPARα [64]. Thus, increased expression of malonyl-CoA decarboxylase is expected to result in lowering of malonyl-CoA levels and, consequently, enhanced mitochondrial fatty acid β-oxidation. Moreover, activated AMPK may also promote fatty acid oxidation through increased delivery of FA substrates via upregulation of hepatic CD36 expression [65].

Additional studies are planned to further delineate the mechanisms by which NDGA improves HFrd-induced hypertriglyceridemia and hepatic steatosis via increased expression of PPARα and activation of AMPK. As noted above, AMPK most likely improves hepatic steatosis and hypertriglyceridemia by inhibiting lipogenesis through increased phosphorylation (inactivation) of ACC and secondarily by promoting hepatic fatty acid oxidation via suppression of malonyl CoA levels. In contrast, our current and previous work suggest that PPARα improves hepatic steatosis and hypertriglyceridemia by promoting increased channeling of fatty acids towards their oxidation and causing the induction of fatty acid oxidation genes; it also to some extent improves hepatic dyslipidemia by interfering with hepatic lipogenesis. However, increasing evidence now suggests that there may be cross talk between AMPK and PPARα and that these two proteins may work in concert to regulate fatty acid oxidation and lipogenesis. For example, activated AMPK is reported to cause catalytic activity-independent transcriptional co-activation of PPARα [66]. Other studies suggest that AMPK may be a down-stream target of PPARα [67], and several studies have shown that PPARα agonists induce AMPKα subunit phosphorylation (activation), the catalytic component of AMPK. Interestingly, our previous studies have shown that NDGA serves as a potent ligand for PPARα [19]. Given this, it is highly likely that NDGA exerts its anti-hyperlipidemic action not only by independently enhancing PPARα expression and activation of AMPK, but also by promoting cross-talk between these two regulatory proteins.

In conclusion, our study shows that treatment with NDGA can attenuate high-fructose diet-induced hypertriglyceridemia and hepatic steatosis (TG accumulation). On the basis of the experimental data obtained, the beneficial actions of NDGA on hypertriglyceridemia and steatosis are exerted by a dual mechanism: inhibition of lipogenesis and enhanced functional expression of the fatty acid β-oxidation pathway. Furthermore, NDGA promotes increased channeling of fatty acids toward oxidation and away from TG synthesis by altering the expression of a number of genes associated with lipid metabolism.

## Supporting Information

S1 Dataset Raw data and statistics. Individual data and statistics for Figs 1–7 and Table 2.dividual data and statistics for Figs 1–7 and Table 2.
(XLS)

## abbreviations

ACC1: acetyl-CoA carboxylase 1
ACOX1: acyl-CoA oxidase 1
Plin2: also known as Adipophilin / ADRP
ARF1: ADP-ribosylation factor 1
AMPK: AMP-activated kinase
ATGL: adipose triglyceride lipase
BSA: bovine serum albumin
CEBPA: CCAAT enhancer binding protein alpha
C/EBPα: CCAAT enhancer binding protein α
CD36: cluster of differentiation 36
CGI-58: comparative gene identification-58
CE: cholesteryl esters
ChREBP: carbohydrate responsive element-binding protein
CMC: carboxymethyl cellulose
CPT1A: carnitine palmitoyltransferase 1A
CPT2: carnitine palmitoyltransferase 2
DGAT1: diacylglycerol-O-acyltransferase 1
DGAT2: diacylglycerol-O-acyltransferase 2
DTT: dithiothreitol; FABP1, fatty acid binding protein 1
FABP4: fatty acid binding protein 4
FASN: fatty acid synthase
FATP5: fatty acid transport protein 5
FC: free-cholesterol
FFA: free-fatty acid
FOXA2: Forkhead box A2
FOXO1: Forkhead box protein O1
G6PC: glucose-6-phosphatase
GCK: glucokinase
GPAT1: glycerol-3-phosphate acyltransferase 1
GSK3: glycogen synthase kinase 3
HFrD: high-fructose- diet
HSL: hormone-sensitive lipase
LCAD: long-chain acyl-CoA dehydrogenase
L-PK: L-type pyruvate kinase
LPL: lipoprotein lipase
L-FABP: liver fatty acid binding protein
LXRα: liver X receptor alpha
LXRβ: liver X receptor beta
MLYCD: malonyl Co-A decarboxylase, MLXIPL, MLX interacting protein-like
MTTP: microsomal triglyceride transfer protein
NDGA: nordihydroguaiaretic acid
PPARα: peroxisome proliferator-activated receptor alpha
PPARγ: peroxisome proliferator-activated receptor gamma
PPARδ: peroxisome proliferator-activated receptor delta
PEPCK: phosphoenolpyruvatecarboxy- kinase
PLD1: phospholipase D1
SCD1: stearoyl-CoA-desaturase 1
SCD2: stearoyl-CoA-desaturase 2
SREBP-1c: sterol regulatory element binding protein-1c
SREBP-2: sterol regulatory element binding protein-2
UPR: unfolded protein response
VLDL-TG: very low-density lipoprotein-triglyceride
TG: triglyceride
XBP1s: X-box binding protein

## References

[1] Chen L, Magliano DJ, Zimmet PZ. The worldwide epidemiology of type 2 diabetes mellitus—present and future perspectives. Nature reviews Endocrinology. 2012;8(4):228–36. Epub 2011/11/09. 10.1038/nrendo.2011.183 . 22064493

[2] Mottillo S, Filion KB, Genest J, Joseph L, Pilote L, Poirier P, et al The metabolic syndrome and cardiovascular risk a systematic review and meta-analysis. Journal of the American College of Cardiology. 2010;56(14):1113–32. Epub 2010/09/25. 10.1016/j.jacc.2010.05.034 . 20863953

[3] Swinburn BA, Sacks G, Hall KD, McPherson K, Finegood DT, Moodie ML, et al The global obesity pandemic: shaped by global drivers and local environments. Lancet. 2011;378(9793):804–14. 10.1016/S0140-6736(11)60813-1 21872749

[4] Ford ES, Li C, Sattar N. Metabolic syndrome and incident diabetes: current state of the evidence. Diabetes care. 2008;31(9):1898–904. 10.2337/dc08-0423 18591398 PMC2518368

[5] Anstee QM, Targher G, Day CP. Progression of NAFLD to diabetes mellitus, cardiovascular disease or cirrhosis. Nat Rev Gastroenterol Hepatol. 2013;10(6):330–44. 10.1038/nrgastro.2013.41 23507799

[6] Lim JS, Mietus-Snyder M, Valente A, Schwarz J-M, Lustig RH. The role of fructose in the pathogenesis of NAFLD and the metabolic syndrome. Nat Rev Gastroenterol Hepatol. 2010;7(5):251–64. 10.1038/nrgastro.2010.41 20368739

[7] Loomba R, Sanyal AJ. The global NAFLD epidemic. Nat Rev Gastroenterol Hepatol. 2013;10(11):686–90. 10.1038/nrgastro.2013.171 24042449

[8] Smith BW, Adams LA. Nonalcoholic fatty liver disease and diabetes mellitus: pathogenesis and treatment. Nature reviews Endocrinology. 2011;7(8):456–65. 10.1038/nrendo.2011.72 21556019

[9] Perry RJ, Samuel VT, Petersen KF, Shulman GI. The role of hepatic lipids in hepatic insulin resistance and type 2 diabetes. Nature. 2014;510(7503):84–91. 10.1038/nature13478 24899308 PMC4489847

[10] Choi SH, Ginsberg HN. Increased very low density lipoprotein (VLDL) secretion, hepatic steatosis, and insulin resistance. Trends Endocrinol Metab. 2011;22(9):353–63. 10.1016/j.tem.2011.04.007 21616678 PMC3163828

[11] Watts GF, Ooi EM, Chan DC. Demystifying the management of hypertriglyceridaemia. Nature reviews Cardiology. 2013;10(11):648–61. Epub 2013/09/26. 10.1038/nrcardio.2013.140 . 24060958

[12] Arteaga S, Andrade-Cetto A, Cardenas R. Larrea tridentata (Creosote bush), an abundant plant of Mexican and US-American deserts and its metabolite nordihydroguaiaretic acid. Journal of ethnopharmacology. 2005;98(3):231–9. Epub 2005/04/09. 10.1016/j.jep.2005.02.002 . 15814253

[13] Bassi N, Karagodin I, Wang S, Vassallo P, Priyanath A, Massaro E, et al Lifestyle Modification for Metabolic Syndrome: A Systematic Review. Am J Med. 2014. 10.1016/j.amjmed.2014.06.035 25004456

[14] Grundy SM. Drug therapy of the metabolic syndrome: minimizing the emerging crisis in polypharmacy. Nat Rev Drug Discov. 2006;5(4):295–309. 16582875 10.1038/nrd2005

[15] Nseir W, Hellou E, Assy N. Role of diet and lifestyle changes in nonalcoholic fatty liver disease. World J Gastroenterol. 2014;20(28):9338–44. 10.3748/wjg.v20.i28.9338 25071328 PMC4110565

[16] Sourianarayanane A, Pagadala MR, Kirwan JP. Management of non-alcoholic fatty liver disease. Minerva gastroenterologica e dietologica. 2013;59(1):69–87. Epub 2013/03/13. . 23478245

[17] Gowri MS, Azhar RK, Kraemer FB, Reaven GM, Azhar S. Masoprocol decreases rat lipolytic activity by decreasing the phosphorylation of HSL. American journal of physiology Endocrinology and metabolism. 2000;279(3):E593–600. Epub 2000/08/19. . 10950827 10.1152/ajpendo.2000.279.3.E593

[18] Kelley GL, Allan G, Azhar S. High dietary fructose induces a hepatic stress response resulting in cholesterol and lipid dysregulation. Endocrinology. 2004;145(2):548–55. Epub 2003/10/25. 10.1210/en.2003-1167 . 14576175

[19] Zhang H, Shen W-J, Cortez Y, Kraemer FB, Azhar S. Nordihydroguaiaretic acid improves metabolic dysregulation and aberrant hepatic lipid metabolism in mice by both PPARα-dependent and-independent pathways. Am J Physiol Gastrointest Liver Physiol. 2013;304(1):72–86. 10.1152/ajpgi.00328.2012 PMC3543637 23104557

[20] Lee M-S, Kim D, Jo K, Hwang J-K. Nordihydroguaiaretic acid protects against high-fat diet-induced fatty liver by activating AMP-activated protein kinase in obese mice. Biochem Biophys Res Commun. 2010;401(1):92–7. 10.1016/j.bbrc.2010.09.016 20836990

[21] Reed MJ, Meszaros K, Entes LJ, Claypool MD, Pinkett JG, Brignetti D, et al Effect of masoprocol on carbohydrate and lipid metabolism in a rat model of Type II diabetes. Diabetologia. 1999;42(1):102–6. 10027587 10.1007/s001250051121

[22] Folch J, Lees M, Sloane Stanley GH. A simple method for the isolation and purification of total lipides from animal tissues. J Biol Chem. 1957;226(1):497–509. Epub 1957/05/01. . 13428781

[23] Mannaerts GP, Debeer LJ, Thomas J, De Schepper PJ. Mitochondrial and peroxisomal fatty acid oxidation in liver homogenates and isolated hepatocytes from control and clofibrate-treated rats. J Biol Chem. 1979;254(11):4585–95. Epub 1979/06/10. . 438207

[24] Yu XX, Drackley JK, Odle J. Rates of mitochondrial and peroxisomal beta-oxidation of palmitate change during postnatal development and food deprivation in liver, kidney and heart of pigs. J Nutr. 1997;127(9):1814–21. 9278565 10.1093/jn/127.9.1814

[25] Scribner KA, Gadbois TM, Gowri M, Azhar S, Reaven GM. Masoprocol decreases serum triglyceride concentrations in rats with fructose-induced hypertriglyceridemia. Metabolism. 2000;49(9):1106–10. Epub 2000/10/04. 10.1053/meta.2000.8604 . 11016888

[26] Hashimoto T. Peroxisomal beta-oxidation enzymes. Cell Biochem Biophys. 2000;32 Spring:63–72. 11330071 10.1385/cbb:32:1-3:63

[27] Miyazaki M, Dobrzyn A, Elias PM, Ntambi JM. Stearoyl-CoA desaturase-2 gene expression is required for lipid synthesis during early skin and liver development. Proc Natl Acad Sci U S A. 2005;102(35):12501–6. 16118274 10.1073/pnas.0503132102 PMC1194914

[28] Saggerson D. Malonyl-CoA, a key signaling molecule in mammalian cells. Annual review of nutrition. 2008;28:253–72. 10.1146/annurev.nutr.28.061807.155434 18598135

[29] Horton JD, Goldstein JL, Brown MS. SREBPs: activators of the complete program of cholesterol and fatty acid synthesis in the liver. J Clin Invest. 2002;109(9):1125–31. 11994399 10.1172/JCI15593 PMC150968

[30] Iizuka K, Bruick RK, Liang G, Horton JD, Uyeda K. Deficiency of carbohydrate response element-binding protein (ChREBP) reduces lipogenesis as well as glycolysis. Proc Natl Acad Sci U S A. 2004;101(19):7281–6. 15118080 10.1073/pnas.0401516101 PMC409910

[31] Strable MS, Ntambi JM. Genetic control of de novo lipogenesis: role in diet-induced obesity. Critical reviews in biochemistry and molecular biology. 2010;45(3):199–214. 10.3109/10409231003667500 20218765 PMC2874080

[32] Xu X, So J-S, Park J-G, Lee A-H. Transcriptional control of hepatic lipid metabolism by SREBP and ChREBP. Semin Liver Dis. 2013;33(4):301–11. 10.1055/s-0033-1358523 24222088 PMC4035704

[33] Wong RH, Sul HS. Insulin signaling in fatty acid and fat synthesis: a transcriptional perspective. Current opinion in pharmacology. 2010;10(6):684–91. Epub 2010/09/08. 10.1016/j.coph.2010.08.004 ; PubMed Central PMCID: PMCPmc3092640. 20817607 PMC3092640

[34] Aoyama T, Peters JM, Iritani N, Nakajima T, Furihata K, Hashimoto T, et al Altered constitutive expression of fatty acid-metabolizing enzymes in mice lacking the peroxisome proliferator-activated receptor alpha (PPARalpha). J Biol Chem. 1998;273(10):5678–84. Epub 1998/04/16. . 9488698 10.1074/jbc.273.10.5678

[35] Kersten S. Integrated physiology and systems biology of PPARα. Mol Metab. 2014;3(4):354–71. 10.1016/j.molmet.2014.02.002 24944896 PMC4060217

[36] Carling D, Thornton C, Woods A, Sanders MJ. AMP-activated protein kinase: new regulation, new roles? Biochem J. 2012;445(1):11–27. Epub 2012/06/19. 10.1042/bj20120546 . 22702974

[37] Hardie DG. AMPK: a target for drugs and natural products with effects on both diabetes and cancer. Diabetes. 2013;62(7):2164–72. 10.2337/db13-0368 23801715 PMC3712072

[38] Munday MR. Regulation of mammalian acetyl-CoA carboxylase. Biochem Soc Trans. 2002;30(Pt 6):1059–64. 12440972 10.1042/bst0301059

[39] Taniguchi CM, Emanuelli B, Kahn CR. Critical nodes in signalling pathways: insights into insulin action. Nat Rev Mol Cell Biol. 2006;7(2):85–96. 16493415 10.1038/nrm1837

[40] Rayasam GV, Tulasi VK, Sodhi R, Davis JA, Ray A. Glycogen synthase kinase 3: more than a namesake. Br J Pharmacol. 2009;156(6):885–98. 10.1111/j.1476-5381.2008.00085.x 19366350 PMC2697722

[41] Guo S. Decoding insulin resistance and metabolic syndrome for promising therapeutic intervention. The Journal of endocrinology. 2014;220(2):1–3. 24431466 10.1530/JOE-13-0584 PMC4068335

[42] Patel S, Doble BW, MacAulay K, Sinclair EM, Drucker DJ, Woodgett JR. Tissue-specific role of glycogen synthase kinase 3beta in glucose homeostasis and insulin action. Mol Cell Biol. 2008;28(20):6314–28. 10.1128/MCB.00763-08 18694957 PMC2577415

[43] Jornayvaz FR, Samuel VT, Shulman GI. The role of muscle insulin resistance in the pathogenesis of atherogenic dyslipidemia and nonalcoholic fatty liver disease associated with the metabolic syndrome. Annual review of nutrition. 2010;30:273–90. 10.1146/annurev.nutr.012809.104726 20645852 PMC3730129

[44] Ussher JR, Koves TR, Cadete VJ, Zhang L, Jaswal JS, Swyrd SJ, et al Inhibition of de novo ceramide synthesis reverses diet-induced insulin resistance and enhances whole-body oxygen consumption. Diabetes. 2010;59(10):2453–64. Epub 2010/06/05. 10.2337/db09-1293 ; PubMed Central PMCID: PMCPmc3279530. 20522596 PMC3279530

[45] Holland WL, Miller RA, Wang ZV, Sun K, Barth BM, Bui HH, et al Receptor-mediated activation of ceramidase activity initiates the pleiotropic actions of adiponectin. Nature medicine. 2011;17(1):55–63. Epub 2010/12/28. 10.1038/nm.2277 ; PubMed Central PMCID: PMCPmc3134999. 21186369 PMC3134999

[46] Monetti M, Levin MC, Watt MJ, Sajan MP, Marmor S, Hubbard BK, et al Dissociation of hepatic steatosis and insulin resistance in mice overexpressing DGAT in the liver. Cell metabolism. 2007;6(1):69–78. Epub 2007/07/10. 10.1016/j.cmet.2007.05.005 . 17618857

[47] Minehira K, Young SG, Villanueva CJ, Yetukuri L, Oresic M, Hellerstein MK, et al Blocking VLDL secretion causes hepatic steatosis but does not affect peripheral lipid stores or insulin sensitivity in mice. J Lipid Res. 2008;49(9):2038–44. Epub 2008/06/03. 10.1194/jlr.M800248-JLR200 ; PubMed Central PMCID: PMCPmc3837456. 18515909 PMC3837456

[48] Magkos F, Su X, Bradley D, Fabbrini E, Conte C, Eagon JC, et al Intrahepatic diacylglycerol content is associated with hepatic insulin resistance in obese subjects. Gastroenterology. 2012;142(7):1444–6.e2. Epub 2012/03/20. 10.1053/j.gastro.2012.03.003 ; PubMed Central PMCID: PMCPmc3564653. 22425588 PMC3564653

[49] Galbo T, Perry RJ, Jurczak MJ, Camporez JP, Alves TC, Kahn M, et al Saturated and unsaturated fat induce hepatic insulin resistance independently of TLR-4 signaling and ceramide synthesis in vivo. Proc Natl Acad Sci U S A. 2013;110(31):12780–5. Epub 2013/07/11. 10.1073/pnas.1311176110 ; PubMed Central PMCID: PMCPmc3732992. 23840067 PMC3732992

[50] Aerts JM, Ottenhoff R, Powlson AS, Grefhorst A, van Eijk M, Dubbelhuis PF, et al Pharmacological inhibition of glucosylceramide synthase enhances insulin sensitivity. Diabetes. 2007;56(5):1341–9. Epub 2007/02/09. 10.2337/db06-1619 . 17287460 PMC4298701

[51] Holland WL, Brozinick JT, Wang LP, Hawkins ED, Sargent KM, Liu Y, et al Inhibition of ceramide synthesis ameliorates glucocorticoid-, saturated-fat-, and obesity-induced insulin resistance. Cell metabolism. 2007;5(3):167–79. Epub 2007/03/07. 10.1016/j.cmet.2007.01.002 . 17339025

[52] Cinar R, Godlewski G, Liu J, Tam J, Jourdan T, Mukhopadhyay B, et al Hepatic cannabinoid-1 receptors mediate diet-induced insulin resistance by increasing de novo synthesis of long-chain ceramides. Hepatology (Baltimore, Md). 2014;59(1):143–53. Epub 2013/07/09. 10.1002/hep.26606 ; PubMed Central PMCID: PMCPmc3839256. 23832510 PMC3839256

[53] Henry PJ. Inhibitory effects of nordihydroguaiaretic acid on ETA-receptor-mediated contractions to endothelin-1 in rat trachea. Br J Pharmacol. 1994;111(2):561–9. 8004399 10.1111/j.1476-5381.1994.tb14774.x PMC1909986

[54] Liu Y, Wang H, Zhu Y, Chen L, Qu Y, Zhu Y. The protective effect of nordihydroguaiaretic acid on cerebral ischemia/reperfusion injury is mediated by the JNK pathway. Brain research. 2012;1445:73–81. Epub 2012/02/14. 10.1016/j.brainres.2012.01.031 . 22325100

[55] Mandard S, Muller M, Kersten S. Peroxisome proliferator-activated receptor alpha target genes. Cell Mol Life Sci. 2004;61(4):393–416. 14999402 10.1007/s00018-003-3216-3 PMC11138883

[56] Anderson CM, Stahl A. SLC27 fatty acid transport proteins. Mol Aspects Med. 2013;34(2–3):516–28. 10.1016/j.mam.2012.07.010 23506886 PMC3602789

[57] Zhang BB, Zhou G, Li C. AMPK: an emerging drug target for diabetes and the metabolic syndrome. Cell metabolism. 2009;9(5):407–16. 10.1016/j.cmet.2009.03.012 19416711

[58] Li Y, Xu S, Mihaylova MM, Zheng B, Hou X, Jiang B, et al AMPK phosphorylates and inhibits SREBP activity to attenuate hepatic steatosis and atherosclerosis in diet-induced insulin-resistant mice. Cell metabolism. 2011;13(4):376–88. Epub 2011/04/05. 10.1016/j.cmet.2011.03.009 ; PubMed Central PMCID: PMCPmc3086578. 21459323 PMC3086578

[59] Kawaguchi T, Osatomi K, Yamashita H, Kabashima T, Uyeda K. Mechanism for fatty acid "sparing" effect on glucose-induced transcription: regulation of carbohydrate-responsive element-binding protein by AMP-activated protein kinase. J Biol Chem. 2002;277(6):3829–35. 11724780 10.1074/jbc.M107895200

[60] Kerner J, Hoppel C. Fatty acid import into mitochondria. Biochim Biophys Acta. 2000;1486(1):1–17. 10856709 10.1016/s1388-1981(00)00044-5

[61] McGarry JD, Brown NF. The mitochondrial carnitine palmitoyltransferase system. From concept to molecular analysis. Eur J Biochem. 1997;244(1):1–14. 9063439 10.1111/j.1432-1033.1997.00001.x

[62] Davies SP, Sim AT, Hardie DG. Location and function of three sites phosphorylated on rat acetyl-CoA carboxylase by the AMP-activated protein kinase. Eur J Biochem. 1990;187(1):183–90. 1967580 10.1111/j.1432-1033.1990.tb15293.x

[63] Assifi MM, Suchankova G, Constant S, Prentki M, Saha AK, Ruderman NB. AMP-activated protein kinase and coordination of hepatic fatty acid metabolism of starved/carbohydrate-refed rats. American journal of physiology Endocrinology and metabolism. 2005;289(5):794–800. 10.1152/ajpendo.00144.2005 15956049

[64] Lee GY, Kim NH, Zhao Z-S, Cha BS, Kim YS. Peroxisomal-proliferator-activated receptor alpha activates transcription of the rat hepatic malonyl-CoA decarboxylase gene: a key regulation of malonyl-CoA level. Biochem J. 2004;378(Pt 3):983–90. 14641110 10.1042/BJ20031565 PMC1224007

[65] Habets DD, Coumans WA, El Hasnaoui M, Zarrinpashneh E, Bertrand L, Viollet B, et al Crucial role for LKB1 to AMPKalpha2 axis in the regulation of CD36-mediated long-chain fatty acid uptake into cardiomyocytes. Biochim Biophys Acta. 2009;1791(3):212–9. Epub 2009/01/23. 10.1016/j.bbalip.2008.12.009 . 19159696

[66] Bronner M, Hertz R, Bar-Tana J. Kinase-independent transcriptional co-activation of peroxisome proliferator-activated receptor alpha by AMP-activated protein kinase. Biochem J. 2004;384(Pt 2):295–305. Epub 2004/08/18. 10.1042/bj20040955 15312046 PMC1134113

[67] Lee WJ, Kim M, Park HS, Kim HS, Jeon MJ, Oh KS, et al AMPK activation increases fatty acid oxidation in skeletal muscle by activating PPARalpha and PGC-1. Biochem Biophys Res Commun. 2006;340(1):291–5. Epub 2005/12/21. 10.1016/j.bbrc.2005.12.011 . 16364253

